# Defining Acute Stress Reactions to support large scale combat operations

**DOI:** 10.1080/08995605.2026.2664947

**Published:** 2026-05-12

**Authors:** Emily G. Lowery-Gionta, Liana M. Matson, Samuel A. McLean, Amy B. Adler

**Affiliations:** a Center for Military Psychiatry and Neuroscience, Walter Reed Army Institute of Research, Maryland, Silver Spring; b Institute for Trauma Recovery, Department of Psychiatry, University of North Carolina at Chapel Hill, Chapel Hill, North Carolina; c Department of Emergency Medicine, University of North Carolina at Chapel Hill, Chapel Hill, North Carolina

**Keywords:** Acute Stress Reaction, trauma, large scale combat operations, military, stress

## Abstract

The United States military anticipates that future warfare will involve large scale combat operations (LSCO). The expected nature of LSCO will likely increase the occurrence and severity of Acute Stress Reactions (ASRs), responses to extreme stressors characterized by cognitive, emotional, somatic/physical, and behavioral symptoms. Conceptualizations of ASR vary, with ASRs defined as a disorder in the 10^th^ version of the International Classification of Diseases (ICD) and considered a non-diagnosable stress reaction by the US military. The diversity in the conceptualizations of ASR has made studying and developing solutions for ASR difficult. Incorporating previous conceptualizations and emerging research, a definition of ASR is proposed that identifies ASR symptom domains, clarifies the timeline in which ASR may occur, and specifies impact of ASR on functioning and neurocognition. To our knowledge, this manuscript is the first to (1) systematically review the current definitions of ASR, (2) specifically discuss ASR in a LSCO context, and (3) present a comprehensive definition of ASR.

## Introduction

The United States (U.S.) military anticipates that future conflicts will involve large scale combat operations (LSCO), defined as resource-intensive joint military operations fought across a full spectrum of battlefield domains, including air, space, land, sea, and cyber (Department of the Army, 2022; Department of the Army, TRADOC, 2024). While the U.S. has previous experience with LSCO, the nature of future LSCO is expected to be uniquely challenging due to technological advances and competition for battlespace that the U.S. has historically controlled (Department of the Army, TRADOC, 2024). For example, the widespread availability of technically sophisticated drones has leveled the competition for control of airspace. The anticipated lack of airspace control in LSCO will impose the requirement for prolonged field care, increase the threat of exposure to enemies, and increase the risk of being attacked. Additional challenges include high operations tempo, lack of access to supply lines, and lack of secure communications (Izaguirre et al., 2025; Marsh & Hampton, 2022). Under these conditions, warfighters will be required to withstand extreme physical and psychological stressors for prolonged periods (Armed Services Biomedical Research, Evaluation and Management (ASBREM) Community of Interest (CoI) [ASBREM], 2017; Defense Health Agency (DHA) & Psychological Health Center of Excellence (PHCoE)S, 2024).

Exposure to such stressors evokes a broad range of natural biological, behavioral, and psychological responses. These responses can be adaptive if they help the individual to function; however, these responses can be maladaptive if they interfere with coping, impair functioning, and are not commensurate to the demands of the stressor. Some responses to extreme stressors can be classified as Acute Stress Reactions (ASRs) (World Health Organization [WHO], 1993, 2019). Though definitions of ASRs vary, they are typically described as a combination of cognitive, emotional, somatic, and behavioral symptoms resulting from exposure to extreme physical or psychological stressors. ASR symptoms emerge during or immediately after exposure to the stressor and typically persist for days afterward but may persist for weeks. Though ASRs may be distressing and cause dysfunction, they are usually not considered life-threatening once exposure to the stressor has ended, and they generally resolve without intervention. However, in high stakes environments like LSCO, ASRs can significantly disrupt operational performance and endanger the lives of service members or their team members, making them a relevant clinical concern.

Currently, published estimates for ASR prevalence during military combat operations come from non-LSCO conflicts. According to these estimates, it appears that at least one in six service members engaged in combat experienced a possible ASR (Adler & Gutierrez, 2022). In LSCO, given the anticipated psychological load from frequent and intense stressors and reduced opportunities for recovery (Izaguirre et al., 2025; Marsh & Hampton, 2022), the prevalence of ASR is likely to be higher. Moreover, the impact of ASR-related disruptions to operational performance is also anticipated to be significant. To maintain and support unit effectiveness, interventions to prevent and manage ASRs are urgently needed. Interventions must be evidence-based and suitable for use during military operations. One barrier to developing such interventions is the lack of a cohesive definition of ASRs.

In this paper, we propose a definition of ASR and discuss the implications of ASRs in LSCO. To support the development of a new ASR definition, we first discuss existing conceptualizations of ASRs by focusing on the seminal definitions provided in the 10th and 11th versions of the WHO’s International Classification of Diseases (ICD) manuals, a classification system used worldwide to identify and record medical conditions (WHO, 1993, 2019). We also discuss the U.S. military’s existing conceptualizations of stress-related conditions including ASR. We then differentiate between ASR conceptualizations and Acute Stress Disorder (ASD), a trauma-related disorder that may emerge during the acute post-trauma timeframe. Finally, we propose a cohesive definition of ASRs that integrates current knowledge and discuss ASRs in the context of LSCO. To our knowledge, this manuscript is the first to (1) systematically review the current definitions of ASR, (2) specifically discuss ASRs in a LSCO context, and (3) present a comprehensive definition of ASR.

## Existing conceptualizations of ASRs

ASRs have been conceptualized in various ways. The main elements of these conceptualizations can be found in Table 1. Given that the differences in conceptualizations are nuanced, direct quotes are used for accurate representation in the table and text. Here, we review ASR conceptualizations from the ICD versions 10 and 11 (WHO, 1993, 2019), the Department of Defense Instruction (DODI) on Combat and Operational Stress Reaction (DoD, 2020), and the “Guideline for Forward Management of Acute Behavioral Health Conditions by Non-Specialty Medical Personnel” (DHA & PHCoE, 2024).

The ICD provides one of the first clinical definitions of ASR. Although earlier versions of the ICD included the concepts of combat neurosis, severe stress reactions, and acute reaction to stress, the 10th version of the ICD (ICD-10) was the first to use the term “Acute Stress Reaction” (Maercker et al., 2013). The ICD-10 was originally published in 1993 and adopted by the U.S. in 2015. The ICD-10 nests ASRs within the “reaction to severe stress, and adjustment disorders” category. This category falls under the broader category of “neurotic, stress-related and somatoform disorders” within the parent category of “mental and behavioral disorders” (WHO, 1993). The ICD-10 defines ASR as a “transient disorder” occurring in the absence of another mental disorder in response to “an exceptionally stressful life event,” also described as “exceptional physical and mental stress.” The ICD-10 considers ASR symptoms to generally emerge during exposure to the stressor and to “disappear” within two to three days, most often “within hours.” Finally, this definition recommends that a different diagnosis be considered “if symptoms persist.” Altogether, this landmark definition conceptualizes ASRs as a diagnosable type of stress disorder with symptoms occurring in response to extreme stressors that are typically transient.

The 11th version of the ICD (ICD-11) became available for widespread use in 2022 and includes substantial changes to the ASR definition (WHO, 2019). In contrast to its classification as a disorder in the ICD-10, ASRs are not classified in the ICD-11 as a disorder but rather as “problems associated with harmful or traumatic events” within the “factors influencing health status” subcategory and the “factors influencing health status or contact with health services” parent category. Indeed, the ICD-11 excludes ASRs from the “disorders specifically associated with stress” category. This distinction is further emphasized in the ICD-11 definition through the statement that stress-related disorders are differential diagnoses. The ICD-11 definition instead describes ASRs as “transient emotional, somatic, cognitive, or behavioral symptoms” stemming from exposure to an “extremely threatening” or “horrific” event or situation. In the ICD-11 definition, ASRs are “considered to be normal.” Importantly, while the ICD-11 acknowledges that ASRs may involve “substantial” distress and impaired functioning, these elements are not essential to the definition. The essential features of the ICD-11 definition are instead: exposure to an “extremely threatening” or horrifying event or situation, the “normal” nature of the response given the stressor, and the dissipation of symptoms “within a few days” if the stressor is removed or at most “approximately 1 month” if the stressor endures. Altogether, the ICD-11 conceptualizes ASRs as normal, non-disordered, and transient responses to extreme stressors. Further, the ICD-11 conceptualizes the duration of ASR symptoms as temporally tied to the presence of the stressor.

Contrasting the two ICD definitions reveals common elements. Both definitions specify that ASRs result from an extremely stressful event. Both definitions also note that symptoms emerge immediately or soon after stress exposure. Additionally, both definitions emphasize that symptoms are typically transient.

At the same time, the ICD definitions have important differences. Most notably, the ICD-10 classifies ASR as a disorder while the ICD-11 does not. With respect to symptom duration, the ICD-10 focuses on the initial 2–3 days without mention of how long symptoms might last beyond that, while the ICD-11 emphasizes the immediate nature of symptoms and the possibility that symptoms may last longer under certain conditions. With respect to symptom types, the ICD-10 describes the course of specific symptoms but does not summarize them by domain. In contrast, ICD-11 suggests specific symptoms and emphasizes that these symptoms fall within the emotional, somatic cognitive, or behavioral domains.

Another way in which the two definitions diverge is the degree to which a link between ASRs and long-term sequelae is discussed. In the ICD-10, consideration of a change in diagnosis is recommended if symptoms persist beyond 2–3 days, and no suggestions for alternative diagnoses are specified. In contrast, the ICD-11 recommends consideration of other disorders (such as adjustment disorder or post-traumatic stress disorder [PTSD]) if symptoms do not subside in the absence of the stressor. Thus, the ICD-11 appears to imply that a continuation of ASR symptoms may reflect the onset of a diagnosable disorder. The ICD-11 also identifies potential differential diagnoses that can result after experiencing a traumatic event (i.e., acute and transient psychotic disorder, depressive disorder, anxiety or fear-related disorders, and dissociative disorders).

A final distinction between the ICD-10 and the ICD-11 is the prominence of distress and impaired functioning. While the ICD-10 states that ASRs can “be regarded as maladaptive responses to severe or continued stress” that “interfere with successful coping mechanisms and therefore lead to problems of social functioning,” the ICD-11 includes distress and impaired functioning as possible but not essential. Thus, the ICD-10 specifies that ASRs are a maladaptive type of stress response, whereas the ICD-11 may include all stress responses (i.e., both adaptive and maladaptive).

The ICD is important to consider because the U.S. military uses the ICD for medical coding. While U.S. clinicians also rely on the Diagnostic and Statistical Manual of Mental Disorders (DSM), the DSM does not include a definition of ASR (American Psychiatric Association, 2022). Even so, the U.S. military recognizes ASRs as a significant psychological concern that may arise during military operations and integrates the concept of ASRs into guidelines for forward management of psychological health.

Thus, beyond the ICD-10 and ICD-11 definitions, there are additional frameworks within which ASR exists when considered in the U.S. military context. Specifically, ASRs occurring in military settings may be considered a type of Combat and Operational Stress Reaction (COSR). As defined by the Department of Defense (DoD) in DoD Instruction (DoDI) 6490.05, “Maintenance of Psychological Health in Military Operations,” COSRs are physical, emotional, cognitive or behavioral reactions, adverse consequences or psychological injuries experienced by Service Members in response to stressful or traumatic combat events or military operations (DoD, 2020). Notably, DoDI 6490.05 does not provide a list of possible COSR symptoms, nor does it delineate the possible duration of COSRs. Instead, COSR is an umbrella designation under which many stress-related responses, including those of ASR, may fall. Importantly, consistent with the ICD-11 definition of ASRs, COSRs are by definition not diagnosable medical or mental health disorders. Likewise, COSRs do not necessarily entail disrupted functioning though the description implies impairment.

There are two primary differences between the ICD-defined ASR and the DoDI-defined COSR conceptualizations. First, ICD-defined ASRs can occur in response to any type of extreme stress exposure, whereas COSRs are specific to combat and trauma-related stressors or other military operational stressors (e.g., isolation, environmental stress, family stress, and other non-combat stressors associated with deployment). Second, ASRs are generally transient (resolving in less than a month). In comparison, no timeframe is specified for COSRs by DoDI 6490.05.

Although ASRs are not mentioned by name in the DoDI’s description of COSR, they are described in a recent Joint Trauma Surgeon Guideline. Specifically, the “Guideline for Forward Management of Acute Behavioral Health Conditions by Non-Specialty Medical Personnel” (henceforth called the Guideline) addresses ASRs within military operations (DHA & PHCoE, 2024). Notably, the Guideline relies on the ASR definition from the ICD-10 (not the ICD-11). However, unlike the ICD-10 definition, the Guideline is silent on whether ASRs are a diagnosable condition. As defined by the Guideline, ASRs are “involuntary, temporary reactions that overwhelm [a service member’s] ability to function” due to “physical, cognitive, behavioral and emotional effects.” This definition indicates that ASRs stem from experiencing “exceptional” mental or physical stressors occurring in life-threatening environments. Additionally, ASRs are noted to last up to 48 hours after stressor exposure and “to become a problem when they stop service members from functioning,” especially in the context of combat. Thus, the Guideline is concerned with ASRs when operational performance is disrupted and highlights the importance of thinking about ASRs in the context of combat operations.

Altogether, the existing conceptualizations of ASR characterize ASRs as responses to extreme stressors with symptoms that are generally transient. All conceptualizations acknowledge that ASRs are constituted by a variety of symptoms and frame ASRs as natural or normal responses. Additionally, all conceptualizations except the ICD-10 emphasize that ASRs are not considered diagnosable disorders. Given the agreement on these definitional elements, they may be considered essential features of ASRs that should be retained in a cohesive definition. Notable areas of divergence across conceptualizations include symptom duration, specificity in the type of stressor that precipitates an ASR (with military sources emphasizing military stressors), and whether ASRs encompass all stress responses or just those that are associated with dysfunction or maladaptation. These areas of disagreement require congruence or clarification to develop a cohesive definition of ASRs.

## Understanding the scope of ASR symptoms

An understanding of possible ASR symptom presentations is critical to forming a cohesive definition of ASRs, especially one that is useful in military contexts. Here, we review possible ASR symptoms found in the 10th and 11th versions of the ICD and the Guideline, as well as in recent research studies in military and civilian populations (Adler & Gutierrez, 2022; Adler et al., 2020; Lowery-Gionta et al., 2025). A diagram of possible ASR symptoms using the language from each primary source can be found in Supplemental Figure S1.

All existing ASR conceptualizations include descriptions of possible ASR symptoms. The ICD-10 notes that ASR symptoms may follow a particular course beginning with being in a dazed state then moving to either a withdrawn state or an agitated state. Other symptoms noted in the ICD-10 include amnesia and “autonomic signs of panic anxiety” like tachycardia, sweating or flushing. According to the ICD-11, examples of possible symptoms include “autonomic signs of anxiety,” “being in a daze,” confusion, sadness, and anger. Additional symptoms include overactivity or inactivity, social withdrawal, and stupor. It is noted that depersonalization, derealization and amnesia may also be observed. The Guideline (DHA & PHCoE, 2024) suggests possible ASR symptoms such as palpitations or pounding heart, sweating, trembling or shaking, chest pain or discomfort, nausea or stomach distress, and feeling dizzy, unsteady, faint or light-headed. Fear of losing control, going crazy or passing out, and fear of dying are also included as other possible symptoms.

With respect to research, surveys conducted with U.S. Army soldiers illuminate the types of symptoms that may occur during possible ASRs associated with military operations (Adler et al., 2020). For example, soldiers with combat deployment experience who had observed possible ASRs in another soldier reported observing symptoms such as inability to function, detachment, being emotionally overwhelmed, extreme confusion, and being erratic and agitated. Nearly one third of soldiers reported that they felt the individual experiencing a possible ASR was a risk to themselves and others. Results of follow-on studies also highlight the potential impact of ASRs on the ability to function (Adler & Gutierrez, 2022). For example, over half of soldiers who had experienced a possible ASR reported their ability to function was degraded for 5 minutes or longer. Moreover, a small proportion reported that their ability to function was degraded for at least 3 days. Altogether, these lines of evidence suggest that ASRs in combat operations manifest in a variety of symptoms that degrade functioning, thus increasing risk to the individual and unit.

In addition to these retrospective reports from combat veterans, complementary real-time data regarding ASR symptoms have been obtained from civilians. The Advancing Understanding of RecOvery after traumA (AURORA) study conducted assessments of thousands of civilian trauma survivors seeking care at U.S. hospital-based emergency departments (McLean et al., 2020). Lowery-Gionta et al. (2025) analyzed the AURORA data and identified symptoms reported by emergency department patients that may be associated with an ASR, including somatic symptoms (e.g., pain, dizziness, and nausea), cognitive symptoms (e.g., difficulty concentrating, and changes in perception like feeling as if in a dream or as if watching oneself), emotional symptoms (e.g., nervous, worried or anxious; feeling down, no good or worthless; severe anxiety or panic), and behavioral symptoms (e.g., jumpy or easily startled; super alert, watchful or on guard; irritability or having angry outbursts or acting aggressively). Additional symptoms observed at follow-up included fear memory-related symptoms (e.g., avoidance of thoughts, memories, feelings or reminders of the traumatic event; reexperiencing; rehashing) and sleep-related symptoms (e.g., insomnia and nightmares). Some of these symptoms were measured in the first two weeks post-trauma, so validation of these symptoms in the hours and days following stress exposure is needed to better characterize an “acute” response. Nonetheless, results from the AURORA study point to a broad array of symptoms that may constitute an ASR.

Interesting points for consideration arise when examining the range of ASR symptoms identified by these military and civilian surveys. First, the research findings of Lowery-Gionta et al. (2025) validate many of the symptoms delineated in the ICD-10 and ICD-11. This cross-validation is an important step in grasping the many ways in which ASRs can present. Second, Lowery-Gionta et al. (2025) demonstrated that ASRs incorporated a much broader range of symptoms than those provided by the ICD definitions, though as mentioned above, the acute timing of these symptoms must still be examined.

Differences in the degree to which the emotional, physical/somatic, cognitive, and behavioral symptom domains are emphasized within each ASR conceptualization are evident. For example, the ICD-10 lists a total of nine symptoms, most of which are cognitive. Specifically, this list includes seven cognitive symptoms, one behavioral symptom, and one somatic symptom; no emotional symptoms are described. The ICD-11 lists 14 discrete symptoms across a range of domains. This list includes five cognitive symptoms, four emotional symptoms, four behavioral symptoms, and one somatic symptom. The Guideline (DHA & PHCoE, 2024) lists 12 symptoms that are primarily physical. Specifically, this list includes nine physical symptoms, and only two emotional symptoms and one cognitive symptom; no behavioral symptoms are mentioned.

In research publications, the range of symptom domains is similarly varied. Adler et al. (2020) report on six symptoms. They list three behavioral symptoms, two cognitive symptoms, and one emotional symptom; no somatic symptoms are described. In contrast, Lowery-Gionta et al. (2025) assessed a wide array of outcomes and identified 31 possible symptoms. They identified eleven somatic, eight cognitive, six emotional, and six behavioral symptoms. The contrast between Adler et al. (2020) and Lowery-Gionta et al. (2025), especially with respect to somatic symptoms, may reflect difference in methodology. Specifically, Adler et al. (2020) asked soldiers about what they had seen in others and were therefore limited to surveying what could be externally observed, while Lowery-Gionta et al. (2025) studied individual self-reports of internal experiences.

Overall, several notable points emerge from reviewing the current understanding of possible ASR symptoms. First, it is notable that ASR symptoms can, and likely do, interfere with the ability to function. This is interesting, especially in light of the ICD-11’s statement that dysfunction is probable but not necessary in ASRs. Nonetheless, all ASR conceptualizations (including the ICD-11) as well as research findings highlight symptoms that are likely to disrupt the ability to think or act because of overwhelming emotions, intense physical sensations, or altered perceptions of reality. It therefore follows that reduced capacity to continue functioning during an ASR is expected, even though ASRs are normal reactions to extreme stressors. Second, given the many varying ASR symptoms, it is also notable that ASRs can present in a multitude of ways. Finally, as shown Supplemental Figure S1, it is notable that there is substantial convergence across existing conceptualizations and emerging research about what types of symptoms may constitute an ASR. This suggests consistency in what constitutes an ASR despite the differences in how it has been described and defined. Research is needed to determine if symptoms that are commonly represented across sources should be considered hallmarks of ASRs.

## Contrasting ASRs with ASD

It is important to acknowledge that some elements of ASR conceptualizations, as well as some ASR symptoms, overlap substantively or are identical to those of other conditions arising from exposure to extreme stressors or trauma such as ASD and PTSD. Like ASRs, ASD and PTSD can originate from exposure to extreme stressors (Yehuda et al., 1998). From a military perspective, given that extreme stressors are encountered during military operations, each of these conditions is of concern to the U.S. military, both generally and in preparation for LSCO. It is therefore worthwhile to consider the similarities and differences between ASR, ASD, and PTSD.

The key distinction between ASR, ASD, and PTSD is timeframe. As noted previously, ASR conceptualizations generally agree on the acute nature of symptoms. In the case of ASD, symptoms must last at least three days, and in the case of PTSD, symptoms must last at least 30 days in order for these diagnoses to be considered. Thus, ASRs and ASD have some temporal overlap, whereas there is no known temporal overlap between ASRs and PTSD. Given the potential temporal overlap between ASR and ASD, from here forward, we focus on contrasting these conditions. As the ICD does not include a definition of ASD in either the 10th or 11th versions, we will focus our discussion on the ASD definition described in the 5th edition of the DSM (DSM-V).

According to the DSM-V, ASD is a disorder arising from “actual or threatened death, serious injury, or sexual violation” through direct experience or in-person witnessing of the traumatic event, learning of an actual or threatened violent or accidental death of a family member or friend, or repeated or extreme exposure to disturbing details of traumatic events (excluding non-work related media sources) (American Psychiatric Association, 2022). Thus, according to this definition, ASD stems from the stress of specific traumatic experiences-death, injury, or sexual violation. For ASD to be diagnosed, nine or more symptoms must persist for at least three days and up to one month after the traumatic event. Symptoms fall into the following five categories: intrusion symptoms, negative mood symptoms, dissociative symptoms, avoidance symptoms, and arousal symptoms. For the diagnosis to be applied, individuals must experience “clinically significant distress” or impaired ability to function. Though the diagnosis requires symptom persistence for at least three days and up to one month, it is noted that symptoms “typically begin immediately after the trauma” (American Psychiatric Association, 2022).

Similarities in how ASD and ASR are described are evident between the DSM-IV ASD definition and all ASR conceptualizations (American Psychiatric Association, 1994). For example, the DSM-IV ASD definition and all ASR conceptualizations require the onset of symptoms provoked by a specific exposure. Thus, ASR and ASD share the requirement for a discrete point of origin of the condition. The ASD definition and all ASR conceptualizations also imply the requirement of exposure to an extremely stressful, threatening, or traumatic stimulus, event, or situation. Therefore, ASD and ASR are both considered stress-related conditions.

Despite these similarities, differences between the ASD definition and ASR conceptualizations are also evident. For example, the extent of detail provided in the descriptions of what constitutes the precipitating event differ between each ASR conceptualization and the ASD definition. Generally, ASR conceptualizations are less prescriptive and particularized about what stressful circumstances may be the source of symptom onset (although military conceptualizations highlight military stressors), while the ASD definition is more thorough and finite in describing the type of exposure that must be experienced to meet diagnostic requirements. The ASD definition is also more definitive in its requirement of distress or dysfunction, the experience of which some ASR conceptualizations do not explicitly require.

The way that symptoms are described is another clear distinction between ASR conceptualizations and the ASD definition. The ASD definition provides specific symptom categories with detailed descriptions of specific symptoms as well as a requirement for the number of symptoms that must be observed. Thus, ASD is diagnosed only when a specified number of symptoms are present. In contrast, though most ASR conceptualizations define specific symptom domains (i.e., behavioral, cognitive, emotional, and somatic), no ASR conceptualizations define the specific symptoms (or number thereof) that constitute an ASR. Instead, ASR conceptualizations provide some suggested symptoms that may occur without comment on the number of symptoms that may be present. ASR conceptualizations and the ASD definition also differ in the description of symptom duration. ASD symptom duration is strictly defined within a discrete post-exposure window of three days to one month, while ASR symptom duration is more vague as defined by ICD-11 although it emphasizes the immediate period during and following the stressor.

Examination of the specific symptoms constituting an ASD versus those that may occur during an ASR further reveals similarities and differences between these conditions. Many of the symptoms described in the ASD definition overlap with those suggested for ASRs. Shared symptoms mainly relate to amnesia or being dazed, negative feelings or mood, sleep issues, avoidance, hyperarousal, anger, and perceptual distortions of reality. Given the extent of shared symptoms, it may be that symptoms present during an ASR contribute to the development of ASD or reflect emergent ASD; however, research is needed to assess these possibilities. Despite these areas of overlap, many symptoms are not shared. For example, ASRs include somatic symptoms like pain, headache, autonomic activation, difficulty breathing, trembling or shaking, dizziness, and nausea. In contrast, somatic symptoms denoted in ASD are limited to arousal symptoms. Likewise, some cognitive symptoms of ASR, like confusion and limited attention, are not explicitly included in the ASD definition, which focuses more on fear memory responses. In addition, ASR emphasizes emotional symptoms like anxiety, panic, and being overwhelmed, none of which are included in the ASD definition. Thus, ASRs have unique symptoms that may enable their distinction from ASD. It would be interesting to understand through future research how those symptoms of ASR that are shared with ASD, as well as those that are unique, contribute to later psychological health outcomes like development of ASD and/or PTSD.

Overall, it is evident from comparing ASR and ASD that these conditions have common attributes but are distinguishable conditions. The manner in which they are described reveal points of distinction, as do the symptoms that constitute each condition. Furthermore, it is interesting to consider the way that ASR conceptualizations are constructed in comparison with the ASD definition, especially in light of the goal of developing a cohesive ASR definition. Specifically, ASR is broadly defined, while the definition of ASD is definitive and criteria-driven. This contrast may arise from ASD being a diagnosable disorder and ASR being a factor rather than a diagnosis. The open-endedness of ASR conceptualizations and resulting flexibility it affords is thus worthwhile to preserve in a cohesive definition of ASR.

## Defining ASRs for military applications

A cohesive definition of ASR that draws on existing conceptualizations is needed to advance understanding of ASRs and how to manage them. Indeed, a useful definition of ASR should strike a balance between definitiveness and open-endedness to support accurate identification and management. The definition should be broad enough to capture the scope of what may be observed or experienced during an ASR but specific enough that distinctions can be made from other psychological health emergencies. Moreover, a useful definition would be applicable to ASRs experienced under a variety of conditions and across most contexts, including civilian and military settings. With respect to the unique needs of military operations, particularly those that involve LSCO, an ASR definition that is useful would acknowledge ASRs and their impact without pathologizing them. In addition, a definition of ASR that is useful in future LSCO would need to be unambiguous because the responsibility for rapidly identifying and effectively managing ASRs will primarily fall to peers (i.e., other service members) or to non-specialized medical personnel (i.e., medics) who are unlikely have extensive expertise in managing psychological health-related conditions.

To this end, we identified seven main elements that should be incorporated into in a definition of ASR. These elements are: 1) a description of the stressors precipitating ASRs; 2) the involuntary nature of ASRs; 3) the symptom domains affected by ASRs; 4) the impact of ASRs on functioning; 5) the duration of ASRs; 6) the distinction between disorder and condition; and 7) the consideration of follow-on diagnoses. Notably, no single existing ASR conceptualization captures all of these seven key elements (see Table 1).

First, it is important to describe the kinds of stressors that precipitate ASRs. A key feature of all ASR conceptualizations is that extreme stressors are the point of origin for ASRs. Here, we specify that ASRs are prompted by experiences that are extremely threatening or horrific. This description helps to differentiate ASRs from other conditions that may present similarly but without a precipitating experience, such as panic or acute agitation. This description also differentiates ASRs from other conditions that are caused by stressors that are not threatening or horrific, such as adjustment disorder.

Second, we recommend specifying that ASRs are an involuntary, natural response. Historically, it was inaccurately assumed that adverse reactions to stressful or traumatic events like combat reflected a failure of fortitude (Chermol, 1985). By describing ASRs as involuntary and natural responses, the proposed definition may counter this stigma. As a result, service members may have less concerns about their ability to control their reactions to the psychological demands of combat and may also be more open to learning how to help their teammates who experience an ASR. This element also captures the spontaneous emergence of ASRs, and thereby conveys that ASRs could happen to anyone who experiences an extreme stressor. Finally, specifying ASRs as involuntary and natural is consistent with a growing body of evidence that a rapid response to threat can occur without conscious awareness, as with those that bypass cortical processing via “low-road” subcortical pathways to the amgydala (LeDoux, 1996; Wang et al., 2023).

Third, we recommend specifying that ASRs are not a clinical disorder. This designation aligns with the most recent conceptualizations of ASR in the ICD-11 and is consistent with the inclusion of ASR as a type of COSR. This language is also consistent with the description shared by all existing ASR conceptualizations of ASRs as transient, since disorders are typically diagnosed because symptoms persist for substantial periods of time. In addition to reflecting these converging conceptualizations of ASR, another benefit of avoiding language that implies a disorder is that ASRs are not unnecessarily pathologized. Finally, although ASR and ASD may temporally overlap and have some shared symptoms, this language helps to reinforce that ASRs are distinct from ASD. Of note, this designation does not preclude the need to consider differential diagnoses or the subsequent development of disorders over time. This designation also does not preclude the need to mitigate or provide interventions for ASRs, particularly in high stakes contexts like military operations in which ongoing performance is required.

Fourth, we propose describing ASRs as comprised of somatic, emotional, cognitive and/or behavioral symptom domains. The inclusion of these domains is common to recent ASR conceptualizations (i.e., the Guideline and the ICD-11) and coincides with the findings of Lowery-Gionta et al. (2025). Though some definitions use the term “physical” in place of somatic, we opted to use the term somatic to connote the mind-body connection. This phrasing reflects the idea that ASR symptoms can be experienced as bodily sensations coinciding with emotional responses, originating from central nervous system (CNS) activation in response to threats (Herman et al., 2016; McEwen, 2017).

Fifth, to address the potential impact of ASR symptoms, we recommend including a statement that ASRs are accompanied by impaired functioning and neurocognition. Such impairment can range from difficulties in basic functioning (e.g., inability to move) to difficulties in higher order cognitive functioning (e.g., rapid decision-making). There are benefits to including impaired functioning and neurocognition as an element in the definition of ASR, especially one that is useful for military applications. Adding this element signals the need to consider the potential impact of ASRs on accomplishing operational tasks and the concomitant risk of harm to the individual and their unit. Though ASRs may only last for a very short time (e.g., 1–2-seconds), the longer an ASR lasts, the more likely it is to impede functioning in a way that endangers the individual and, in military operations, their team. Thus, adding functioning to the definition raises the possibility that impaired functioning due to ASRs may need to be managed during ongoing operations. Altogether, the display of possible ASR symptoms coinciding with functional and/or neurocognitive impairments together indicate the occurrence of an ASR. Conversely, the display of possible ASR symptoms in the absence of functional and/or neurocognitive impairments would not indicate the occurrence of an ASR, but rather an acute stress response.

The addition of a statement about functioning is in keeping with ICD-11’s designation of ASRs as a “problem” associated with a harmful or traumatic event and with the implied adverse effects on functioning reflected in all other conceptualizations. This addition also clarifies the part of the Guideline that states that ASRs become a problem when service members are unable to function (DHA & PHCoE, 2024). However, it is a departure from the ICD-11, which states that dysfunction is not an essential feature (WHO, 2019). Altogether, the addition of impaired functioning and neurocognition to the definition is a significant step in unifying ASR conceptualizations and communicating the importance of ASRs as a risk factor in military operations.

While it is important to acknowledge that individuals can have a range of stress responses (which can be both adaptive and maladaptive) to an extremely stressful experience, here we are defining ASRs as comprised of stress response symptoms that disrupt functioning. This description clarifies inconsistencies in existing ASR conceptualizations. We note that the adaptive nature of stress responses is dependent on the context in which they are experienced. For example, hypervigilance may be adaptive in some contexts (e.g., during operations in an active conflict zone, hypervigilance can inform decision-making) but maladaptive in others (e.g., upon return to a safe area, hypervigilance can disrupt sleep). Likewise, avoidance may be adaptive in some contexts (e.g., running for cover in response to the sound of an enemy drone) or maladaptive (e.g., running for cover in response to any engine sound). Thus, symptoms in and of themselves are not indicative of an ASR but rather symptoms associated with impaired functioning.

This distinction is an important consideration for the military. During military operations, medics at the forefront of managing ASRs will rely on signs of disrupted function as a signal to intervene. Typically, symptoms of ASR following a mission are not considered adaptive because the individual is in a safer location. However, in a LSCO environment, individuals returning from a mission may still be in an unsafe context. Thus, the LSCO context and high-stakes nature of the military occupation makes this conceptualization different from a civilian in a motor vehicle collision whose enduring stress response would not be adaptive once the immediate danger has passed.

Sixth, to describe the timeframe in which ASR symptoms arise and persist, we recommend that the duration of symptoms be described as transient and specified as occurring within 0–72 hours of the precipitating event. Further, we recommend clarifying that symptoms generally arise during or within hours of the precipitating event. This recommendation remains consistent with existing definitions that describe ASRs as “temporary” or “transient,” as well as those that mention symptom duration as typically no longer than 2–3 days. Importantly, this description is definitive rather than open-ended, thus enabling more accurate identification, tracking, and investigation of ASRs. This description is supported by the observation in service members that ASR symptoms causing functional impairment resolve within a 3-day period in more than 85% of cases (Adler & Gutierrez, 2022). This timeframe also enables temporal distinction of ASRs from ASD and is consistent with the military’s current practice of making return to duty decisions or evacuating to higher roles of care after 72 hours (Department of the Army, 2019).

Seventh, while the proposed definition emphasizes resolution of symptoms within 72 hours, we recommend stating that symptoms may persist beyond 72 hours. If symptoms persist beyond 72 hours, other diagnoses and follow-on interventions may need to be considered. This definitional element acknowledges the potential for longer-term challenges associated with ASRs. We recommend that evaluation for other conditions be considered when symptoms persist, especially in the absence of ongoing or new stressors. This recommendation is important to include in a military-relevant definition because other conditions may have specific requirements or be triaged differently.

Taking these elements into account, we propose the following definition of ASR:

An involuntary, natural stress reaction to an extremely threatening or horrific experience. The reaction does not constitute a medical disorder but is characterized by somatic, emotional, cognitive and/or behavioral symptoms that impair an individual’s functioning and neurocognition. Symptoms and impaired functioning occur during or within hours of the precipitating event or situation and generally recede over the course of 72 hours. When symptoms and impaired functioning persist beyond 72 hours, evaluation for other stress-related conditions should be considered, especially in the absence of ongoing stressors or new stressors.

## Meeting the challenge of ASRs in LSCO

The LSCO context has implications for ASRs. It is anticipated that LSCO will include mass and precision fires, information warfare, contested logistics, and an overall increased risk of lethality (Department of the Army, TRADOC, 2024). Independently, each of these conditions can contribute to more frequent and more intense exposures to stress and therefore have the potential to increase the occurrence, duration, and intensity of ASRs in service members. Service members may also experience multiple extreme stressors or extreme stressors that last for prolonged periods (Marsh & Hampton, 2022). When these conditions occur in combination, the risks of ASR and their adverse impact on service member functioning and neurocognition will likely compound. These risks of ASR will be further exacerbated by minimal opportunities for recovery and limited access to specialized care. Under these conditions, ASRs will need to be identified and managed primarily in theater by non-medically trained service members or medics using immediately available resources.

Traditionally, Combat and Operational Stress Control (COSC) personnel manage and treat behavioral health concerns like ASRs in the field (DOD, 2020). These providers are trained in behavioral health and travel to various locations in order to support multiple units during military operations. This model requires control over the battlespace because COSC personnel need flexible movement, sustained communications, and access to medical supplies. However, in LSCO, these battlefield conditions are unlikely. Tele-health solutions have been proposed to overcome these challenges, although obstacles to secure communication will make remote assistance via tele-healthcare or consultation with specialist providers difficult. Therefore, it will be critical to train unit medics in the basics of ASR identification and management. Training service members on these basics may also be critical as a way to provide “buddy aid” solutions.

It will also be important for leaders at all levels to understand the risk of ASRs to individual and unit effectiveness so that they can adequately plan for the possible impact of ASRs on LSCO. At the strategic level, attrition due to ASRs can be factored into force readiness projections. Guidance for mitigating ASRs and triaging to higher levels of care can also be established. At the tactical level, it would be helpful to develop, refine, and implement strategies for managing ASRs. Plans for detecting and delivering care under the resource constraints of LSCO should also be integrated into training. At the operational level, leaders should ensure that unit members are prepared to identify and manage ASRs.

In addition to education about ASRs, ubiquitous implementation of tools that are currently available to manage ASRs is recommended. The Guideline suggests intervening with the iCOVER intervention as a first-line approach to managing ASRs in theater (DHA & PHCoE, 2024). This brief buddy aid tool is designed to rapidly bring service members experiencing an ASR back to functioning. The U.S. Military and its allies have begun integrating the iCOVER intervention into routine training. Overall, iCOVER has been well-received by service members. Evidence of this intervention’s utility includes feedback from Ukrainian medics trained in iCOVER, who report that this training is relevant to combat in the trenches of Ukraine (Iversen & Adler, 2023), a conflict which may serve as a model for what future LSCO will entail (Crombe & Nagl, 2023).

## Future directions

While initial dissemination of knowledge and tools can be achieved, there are gaps that should be addressed to meet the full scope of ASR-related challenges anticipated in LSCO. First, it is necessary to have a triage system for ASRs. To implement an effective triage system, a greater understanding of ASR recovery trajectories is needed. In the context of LSCO, it will be important to support unit members’ operational performance and avoid unnecessary evacuation, especially in the context of limited access to evacuation capabilities. Thus, knowledge about the severity and duration of ASR symptoms during military operations and how these symptoms relate to recovery of functioning is needed. This knowledge can support development of methods and tools for predicting severe or prolonged ASR-related dysfunction that can inform triage, healthcare delivery, and evacuation decisions.

The relationship between ASR and traumatic brain injury (TBI) is another area that would benefit from further study. Objective identification of TBI can now be performed with the use of highly sensitive blood-based biomarkers obtained in the peritraumatic period (Calcagnile et al., 2016; Luoto et al., 2017; Okonkwo et al., 2013; Papa et al., 2024; Wang et al., 2021). This advancement in the field can help to determine the degree to which ASR and TBI symptoms overlap. Indeed, TBI-specific symptoms or other characteristics displayed by individuals who have biomarker-identified TBI have not been investigated, as far as we know. Furthermore, associations between biomarker-identified TBI symptoms and ASR symptoms are unknown. To our knowledge, only one study has evaluated acute psychological symptoms in biomarker-identified TBI. In this large-scale study of non-hospitalized motor vehicle collision patients presenting to the emergency department, individuals with biomarker-identified TBI (i.e., those expressing high levels of the biomarker GFAP) did not have higher levels of acute and chronic psychological distress and ASR-related symptoms than individuals without biomarker-identified TBI (McLean et al., 2024, 2025). These data suggest that stress-related symptoms that occur during ASR may not be a feature of TBI itself, though more work is needed in this area.

Another gap that must be addressed is interventions for managing ASRs. Currently, iCOVER is the only first-line intervention for ASRs. Given the lack of information about iCOVER’s effectiveness in LSCO, other solutions must be developed. The development of additional solutions would facilitate a stepped care approach to ASR management (Matson et al., 2022). In a stepped care model, the first intervention would be iCOVER. If symptoms are not sufficiently resolved and functioning is not adequately restored, administration of medications would follow. Although there are no FDA-approved medications to treat ASRs, there are active areas of research to address this need (Matson et al., 2022). If evidence-based clinical practice guidelines are developed (or in emergency situations), FDA-approved medications used off-label may fill the current gap in ASR interventions.

An additional gap in knowledge is whether there are cultural variations in ASRs. Since LSCO is expected to be a multi-national effort supported by allies, it is important to consider that stress-related symptoms may be expressed in culturally-specific ways (Backe et al., 2021; Geoffrion et al., 2022). Research is therefore needed to understand how service members of other cultures may experience ASRs. Likewise, materials developed to mitigate ASRs will need to be adapted for use in different cultures. Evidence of the success of this approach comes from the adaptation of iCOVER for allied nations (Adler et al., 2024). Knowledge of ASRs in other cultures will support interoperability between allied militaries.

Current knowledge of the relationships between ASR, ASD, and PTSD suggest that these conditions have complex interactions. However, it remains unclear if experiencing an ASR increases vulnerability to ASD, PTSD, or other disorders. It stands to reason that if ASR is a common response to extreme stressors, its contribution to PTSD development may be minimal given that only a small number of individuals who experience an extremely stressful or traumatic event develop PTSD (Kessler et al., 2017). However, it is important to consider that the nature and strength of relationships between ASR, ASD, and PTSD may be dependent on the type of stressor that is experienced. Indeed, war and combat exposure are stressors that are strongly predictive of PTSD and, consequently, combat veterans are at particular risk for PTSD (Ramchand et al., 2015). Thus, it would be helpful to understand if ASRs experienced during combat impact PTSD development risk differently than ASRs stemming from exposure to other types of extreme stress. Likewise, clear boundaries between ASR and ASD, as well as ASR and PTSD, would be helpful given the notable amount of overlap in symptoms between these conditions. While temporal boundaries have been useful in distinguishing ASD from PTSD, more research is needed to understand if temporal boundaries can be applied to ASR. It is possible that ASR symptoms continue beyond exposure to the extreme stressor or the acute period, especially for exceptional stressors like combat. More information on the longevity of specific ASR symptoms, particularly those that are not also ASD or PTSD symptoms, will further refine the concept of ASR. Altogether, more research is needed to understand how ASRs relate to ASD and PTSD. Research in military contexts would be especially informative because of the unique type of stressors that military service members experience.

Finally, it is important to consider that the current understanding of ASR is based on contexts that may not reflect the relentless nature of psychological demands in LSCO. Specifically, these demands may cause ASR presentations that are more extreme, enduring, or impairing than what has been previously considered. It will therefore be important to continue studying ASR in military contexts, especially in those relevant to LSCO like Ukraine. Depending on future research findings, the definition of ASRs and recommended intervention may need to be reexamined.

By building upon existing conceptualizations of ASRs, we have sought to develop a definition of ASR that is relevant for the military context. Though outstanding questions about ASRs remain, the development of this definition marks an important step toward mitigating the impact of ASRs on service members and their units. This definition offers a foundation for establishing new approaches to managing psychological health during military operations in the context of LSCO.

## Supplementary Material

Supp 1

Supplemental data for this article can be accessed online at https://doi.org/10.1080/08995605.2026.2664947.

## Figures and Tables

**Table 1.** Existing conceptualizations of acute stress reaction.

Elements within Definitions/Conceptualizations	Acute Stress Reaction (ICD-10, 2019)	Acute Stress Reaction (ICD-11, 2025)	Combat and Operational Stress Reaction (DODI 6490.05, 2020)	Acute Stress Reaction (Guideline for Forward Management of Acute Behavioral Health Conditions by Non-Specialty Medical Personnel, 2024)

Controllability	Not mentioned	Not mentioned	Not mentioned	Involuntary
Precipitating event	An exceptionally stressful life event; Exceptional physical and mental stress	An event or situation (either short- or long- lasting) of an extremely threatening or horrific nature	Stressful or traumatic events in combat or military operations	Actual threats to life; exceptional mental or physical stress (e.g., combat)
Medical designation	Classified under neurotic, stress-related and somatoform disorders	Considered normal; not a disorder	Not a mental health disorder or a medically diagnosable condition or concern	Natural response
Domains affected	Specific symptoms mentioned rather than domains	Emotional, somatic, cognitive, or behavioral	Emotional, physical, cognitive, or behavioral	Emotional, physical, cognitive, or behavioral
Relationship to functioning	Not mentioned	May be accompanied by interference with personal functioning	Not mentioned	Reactions become a problem when they stop service members from functioning
Duration	Transient; symptoms appear within minutes and disappear within 2–3 days (often within hours)	Transient; symptoms usually appear within hours to days and usually begin to subside within a few days or following removal from the threatening situation; symptoms may persist when threat is ongoing but are usually greatly reduced within approximately 1 month	Not mentioned	Temporary; 0–48 hours
Long-term outcomes	If symptoms persist, a change in diagnosis should be considered	Symptoms may persist when threat is ongoing	Not mentioned	Higher risk of short-term and long-term psychopathology, such as acute stress disorder, posttraumatic stress disorder, and panic disorder

## Data Availability

This review paper does not include data. Therefore, there is no data available to share.

## What is the public significance of this article?—

Acute Stress Reactions are comprised of cognitive, emotional, somatic/physical, and behavioral symptoms expressed in response to extreme stressors. Many definitions of Acute Stress Reaction have been developed but they differ substantially. This article proposes a definition of Acute Stress Reaction that builds upon previous definitions and focuses on symptoms occurring during or soon after an extremely stressful situation that cause significant dysfunction and impaired neurocognition. The proposed definition will support decision-making during military operations, especially during large scale combat operations in which Acute Stress Reactions are expected to increase in frequency, duration, and intensity.
