# Supplementary material for: Defining Acute Stress Reactions to support large scale combat operations
Source: Mil Psychol. Author manuscript; Available in PMC 2026 Oct 11. (PMC13653637; doi:10.1080/08995605.2026.2664947)
Supplement: Supplementary file 1 [file NIHMS2178959-supplement-Supp_1.docx]

Supplemental Figure 1. Acute Stress Reaction Symptoms by Source.

Caption: Possible Acute Stress Reaction symptoms have been provided by five sources: the ICD-10, the ICD-11, the Guideline, Alder et al. (2020), and Lowery-Gionta et al. (2025). Each source differs in the types of symptoms that are mentioned, highlighting that Acute Stress Reactions can present in many ways. Symptoms that appear in bold are those that are common across the five sources, indicating that they may be core features of Acute Stress Reactions.
